# Supplementary material for: Dexamethasone-loaded platelet-inspired nanoparticles improve intracortical microelectrode recording performance
Source: Nat Commun. 2025 Sep 29;16:8579. doi: 10.1038/s41467-025-63583-z (PMC12480985; doi:10.1038/s41467-025-63583-z)
Supplement: Supplementary file 1 — Supplementary Information [file 41467_2025_63583_MOESM1_ESM.pdf]

Supporting Information

Dexamethasone-loaded platelet-inspired nanoparticles improve intracortical microelectrode recording performance

Longshun Li[1,2], Aniya Hartzler[1], Dhariyat M. Menendez-Lustri[1,2], Jichu Zhang[1], Alex Chen[1], Danny V. Lam[1,2], Baylee Traylor[3], Emma Quill[3], David Nethery[1,2], George F. Hoeferlin[1,2], Christa L. Pawlowski[3], Michael A. Bruckman[3], Anirban Sen Gupta[1], Jeffrey R. Capadona[1,2], Andrew J. Shoffstall[1,2*]

[1]*Department of Biomedical Engineering, Case Western Reserve University, Cleveland, OH, United States*

[2]*Advanced Platform Technology Center, Louis Stokes Cleveland Department of Veterans Affairs Medical Center, Cleveland, OH, United States*

[3]*Haima Therapeutics LLC, Cleveland, OH, United States*

Corresponding Author*

Andrew J. Shoffstall, PhD

Nord Associate Professor | Department of Biomedical Engineering

Case Western Reserve University | Cleveland, OH 44106

ajs215@case.edu | 216-368-1213

Materials and Method Section:

Immunohistochemistry Antibodies Information

Table S1. Immunohistochemistry Antibodies

Target Antigen	1st Antibody	Dilution	2nd Antibody	Dilution
**Neuronal Nuclei (NeuN)**	Mouse anti-NeuN, Millipore Sigma, MAB3477	1:250	Goat anti-mouse AF488, Invitrogen, A32723	1:1000
**Activated Macrophage/Microglia (CD68)**	Mouse anti-CD68, Millipore Sigma, MAB1435	1:100	Goat anti-mouse AF488, Invitrogen, A32723	1:1000
**Glial Fibrillary Acidic Protein (GFAP)**	Rabbit anti-GFAP, Agilent Dako, Z0334429-2	1:500	Goat anti-mouse AF488, Invitrogen, A32723	1:1000
**Immunoglobulin (IgG)**	Rabbit anti-IgG, Bio-Rad, 618501	1:100	Goat anti-rabbit AF488, Invitrogen, A32731	1:1000

Results Section

Characterization of Platelet-Inspired Nanoparticles:

Table S2. PIN Characterization (n = 2, manufacture batches)

Zeta Potential (mV)	Hydrodynamic Diameter (nm)	D90 (nm)
-15.5 ± 3.5	128.0 ± 8.5	160 ± 4.2

Comparisons of the Proportion of Active Electrodes within Treatment Groups at Different Phases:

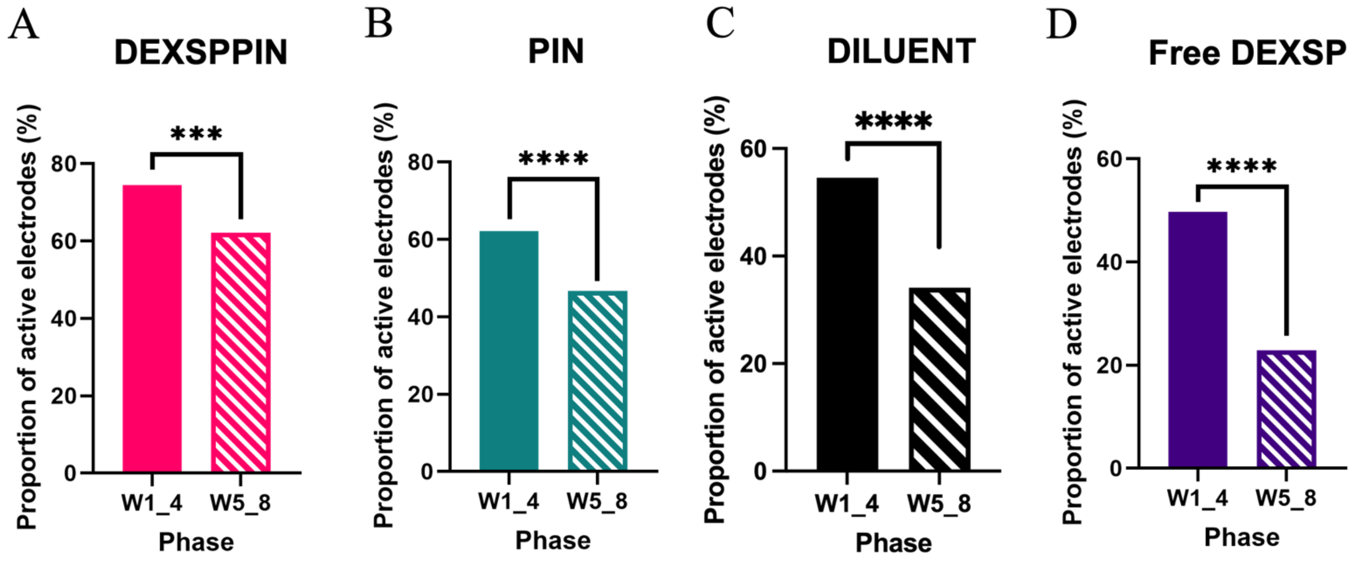

Figure S1. (A-D) Comparisons of the proportion of active electrodes within treatment groups across different phases, indicating a decline in recording performance over time in all groups, with the DEXSPPIN group exhibiting less decline

compared to other groups. The sample size for A-D was determined by the total number of channels multiplied by the number of weeks in each phase and the number of animals in each group: n = 428 for DEXSPPIN, n = 416 for PIN, n = 416 for DILUENT, and n = 472 for Free DEXSP. Channels that were malfunctioning from the beginning were excluded from the analysis.

Full Statistical Comparison for IHC Analysis:

Sample sizes for IHC analysis (number of tissue slices) were as follows: NeuN – DEXSPPIN: 29, Free PIN: 33, TH: 40, Free DEXSP: 33; CD68 – DEXSPPIN: 25, Free PIN: 29, TH: 28, Free DEXSP: 39; GFAP – DEXSPPIN: 27, Free PIN: 32, TH: 26, Free DEXSP: 30; IgG – DEXSPPIN: 24, Free PIN: 26, TH: 30, Free DEXSP: 28. One-way ANOVA analyses with post hoc Tukey pair-wise tests were used to calculate statistical differences within groups for each distance for all biomarkers. Statistical significance was represented as ns = non-significant, * = $p < 0.05$, ** = $p < 0.01$, *** = $p < 0.001$ and **** = $p < 0.0001$ for all tables in this section.

Table S3. Sub-chronic study statistical comparison for NeuN

	DEXSPPIN vs. PIN	DEXSPPIN vs. DILUENT	DEXSPPIN vs. Free DEXSP	PIN vs. DILUENT	PIN vs. DILUENT	DILUENT vs. Free DEXSP
**0-50 µm**	***	****	****	ns	ns	ns
**50-100 µm**	**	***	***	ns	ns	ns
**100-150 µm**	ns	*	***	ns	ns	ns
**150-200 µm**	ns	ns	ns	ns	ns	ns
**200-250 µm**	ns	ns	ns	ns	ns	ns
**250-300 µm**	ns	ns	ns	ns	ns	ns
**300-350 µm**	ns	ns	ns	ns	ns	ns
**350-400 µm**	ns	ns	ns	ns	ns	ns
**400-450 µm**	ns	ns	ns	ns	ns	ns
**450-500 µm**	ns	ns	ns	ns	ns	ns
**500-550 µm**	ns	ns	ns	ns	ns	ns
**550-600 µm**	ns	ns	ns	ns	ns	ns

Table S4. Sub-chronic study statistical comparison for CD68

	DEXSPPIN vs. PIN	DEXSPPIN vs. DILUENT	DEXSPPIN vs. Free DEXSP	PIN vs. DILUENT	PIN vs. DILUENT	DILUENT vs. Free DEXSP
**0-50 µm**	*	*	****	ns	*	*
**50-100 µm**	ns	ns	****	ns	***	****
**100-150 µm**	ns	ns	****	ns	***	****
**150-200 µm**	ns	ns	****	ns	**	****
**200-250 µm**	ns	ns	***	ns	**	***
**250-300 µm**	ns	ns	**	ns	*	**
**300-350 µm**	ns	ns	**	ns	*	***
**350-400 µm**	ns	ns	**	ns	ns	***

**400-450 µm**	ns	ns	**	ns	ns	****
**450-500 µm**	ns	ns	**	ns	*	***
**500-550 µm**	ns	ns	*	ns	ns	**
**550-600 µm**	ns	ns	ns	ns	ns	ns

Table S5. Sub-chronic study statistical comparison for GFAP

	DEXSPPIN vs. PIN	DEXSPPIN vs. DILUENT	DEXSPPIN vs. Free DEXSP	PIN vs. DILUENT	PIN vs. DILUENT	DILUENT vs. Free DEXSP
**0-50 µm**	ns	**	****	*	****	ns
**50-100 µm**	ns	*	**	ns	*	ns
**100-150 µm**	ns	ns	**	ns	*	ns
**150-200 µm**	ns	ns	**	ns	*	ns
**200-250 µm**	ns	ns	**	ns	*	ns
**250-300 µm**	ns	ns	**	ns	*	ns
**300-350 µm**	ns	ns	***	ns	**	ns
**350-400 µm**	ns	ns	***	ns	***	ns
**400-450 µm**	ns	ns	**	ns	**	ns
**450-500 µm**	ns	ns	*	ns	ns	ns
**500-550 µm**	ns	ns	ns	ns	ns	ns
**550-600 µm**	ns	ns	ns	ns	ns	ns

Table S6. Sub-chronic study statistical comparison for IgG

	DEXSPPIN vs. PIN	DEXSPPIN vs. DILUENT	DEXSPPIN vs. Free DEXSP	PIN vs. DILUENT	PIN vs. DILUENT	DILUENT vs. Free DEXSP
**0-50 µm**	ns	*	ns	ns	ns	ns
**50-100 µm**	ns	***	ns	*	ns	ns
**100-150 µm**	ns	***	ns	**	ns	ns
**150-200 µm**	ns	***	ns	**	ns	ns
**200-250 µm**	ns	**	ns	***	*	ns
**250-300 µm**	ns	**	ns	****	**	ns
**300-350 µm**	ns	**	ns	****	**	ns
**350-400 µm**	ns	*	ns	****	***	ns
**400-450 µm**	ns	*	ns	***	**	ns
**450-500 µm**	ns	*	*	**	**	ns
**500-550 µm**	ns	*	*	**	***	ns
**550-600 µm**	ns	ns	*	**	***	ns

Measurement of Creatinine Levels

Table S7. Creatinine Levels Summary

Animal ID	Treatment	Week 0	Week 4	Week 8
**CS12**	DEXSPPIN	< 0.2	0.2	0.2
**CS13**	DEXSPPIN	< 0.2	0.2	< 0.2
**CS14**	DEXSPPIN	0.3	0.2	0.2
**CS22**	DEXSPPIN	0.2	0.2	0.3

**CS23**	DEXSPPIN	0.2	0.2	0.2
**CS24**	DEXSPPIN	< 0.2	0.2	0.2
**CS25**	DEXSPPIN	0.2	0.2	0.2
**CS18**	Free DEXSP	< 0.2	0.2	0.2
**CS29**	Free DEXSP	0.2	0.2	0.2
**CS32**	Free DEXSP	< 0.2	0.2	0.2
**CS42**	Free DEXSP	0.3	0.2	< 0.2
**CS43**	Free DEXSP	0.2	0.2	0.2
**CS48**	Free DEXSP	< 0.2	0.2	0.2
**CS49**	Free DEXSP	0.2	0.2	0.2
**CS50**	Free DEXSP	0.2	0.2	0.2
**CS15**	PIN	< 0.2	0.2	0.2
**CS16**	PIN	0.2	0.2	0.2
**CS17**	PIN	< 0.2	0.2	0.2
**CS40**	PIN	< 0.2	0.3	0.2
**CS45**	PIN	< 0.2	0.2	0.2
**CS46**	PIN	0.2	0.2	0.3
**CS47**	PIN	0.2	0.2	0.2
**CS9**	DILUENT	0.2	0.2	0.2
**CS11**	DILUENT	*	0.2	0.2
**CS19**	DILUENT	0.2	0.2	0.4
**CS21**	DILUENT	< 0.2	0.2	0.3
**CS26**	DILUENT	< 0.2	0.2	< 0.2
**CS27**	DILUENT	< 0.2	0.2	0.3
**CS28**	DILUENT	0.2	0.2	0.2

“*” MISSING: failed to collect blood samples or analysis errors.
