# Supplementary material for: Dexamethasone-loaded platelet-inspired nanoparticles improve intracortical microelectrode recording performance
Source: Nat Commun. 2025 Sep 29;16:8579. doi: 10.1038/s41467-025-63583-z (PMC12480985; doi:10.1038/s41467-025-63583-z)
Supplement: Supplementary file 2 — Reporting Summary [file 41467_2025_63583_MOESM2_ESM.pdf]

nature portfolio

Corresponding author(s): Shoffstall, Andrew

Last updated by author(s): Jun 23, 2025

Reporting Summary

Nature Portfolio wishes to improve the reproducibility of the work that we publish. This form provides structure for consistency and transparency in reporting. For further information on Nature Portfolio policies, see our Editorial Policies and the Editorial Policy Checklist.

Statistics

For all statistical analyses, confirm that the following items are present in the figure legend, table legend, main text, or Methods section.

n/a	Confirmed	
☐	☒	The exact sample size (*n*) for each experimental group/condition, given as a discrete number and unit of measurement
☐	☒	A statement on whether measurements were taken from distinct samples or whether the same sample was measured repeatedly
☐	☒	The statistical test(s) used AND whether they are one- or two-sided<br>*Only common tests should be described solely by name; describe more complex techniques in the Methods section.*
☐	☒	A description of all covariates tested
☐	☒	A description of any assumptions or corrections, such as tests of normality and adjustment for multiple comparisons
☐	☒	A full description of the statistical parameters including central tendency (e.g. means) or other basic estimates (e.g. regression coefficient) AND variation (e.g. standard deviation) or associated estimates of uncertainty (e.g. confidence intervals)
☐	☒	For null hypothesis testing, the test statistic (e.g. *F*, *t*, *r*) with confidence intervals, effect sizes, degrees of freedom and *P* value noted<br>*Give P values as exact values whenever suitable.*
☒	☐	For Bayesian analysis, information on the choice of priors and Markov chain Monte Carlo settings
☒	☐	For hierarchical and complex designs, identification of the appropriate level for tests and full reporting of outcomes
☒	☐	Estimates of effect sizes (e.g. Cohen's *d*, Pearson's *r*), indicating how they were calculated

Our web collection on statistics for biologists contains articles on many of the points above.

Software and code

Policy information about availability of computer code

Data collection	Gamry Echem Analyst v2 was used to collect impedance spectra of the electrodes. TDT Synapse v98 was used to collect electrophysiology data. Zeiss Zen 3 was used for fluorescent slide imaging.
Data analysis	Plexon Offline Sorter and custom Matlab scripts were used as described in the methods section for electrophysiology data analysis. Custom Python scripts ("SECOND") were used to analyze the fluorescent images (available open source, DOI: 10.5281/zenodo.15603085). Microsoft Excel and GraphPad Prism were used for data compilation and plotting. R was used for statistical analysis.

For manuscripts utilizing custom algorithms or software that are central to the research but not yet described in published literature, software must be made available to editors and reviewers. We strongly encourage code deposition in a community repository (e.g. GitHub). See the Nature Portfolio guidelines for submitting code & software for further information.

Data

Policy information about availability of data

All manuscripts must include a data availability statement. This statement should provide the following information, where applicable:

- Accession codes, unique identifiers, or web links for publicly available datasets
- A description of any restrictions on data availability
- For clinical datasets or third party data, please ensure that the statement adheres to our policy

The drug release, electrophysiology, immunohistochemistry, and blood testing data generated in this study have been deposited in a github repository https://github.com/Shofflab/SPPINDEX.

Research involving human participants, their data, or biological material

Policy information about studies with human participants or human data. See also policy information about sex, gender (identity/presentation), and sexual orientation and race, ethnicity and racism.

Reporting on sex and gender	n/a
Reporting on race, ethnicity, or other socially relevant groupings	n/a
Population characteristics	n/a
Recruitment	n/a
Ethics oversight	n/a

Note that full information on the approval of the study protocol must also be provided in the manuscript.

Field-specific reporting

Please select the one below that is the best fit for your research. If you are not sure, read the appropriate sections before making your selection.

☒ Life sciences ☐ Behavioural & social sciences ☐ Ecological, evolutionary & environmental sciences

For a reference copy of the document with all sections, see nature.com/documents/nr-reporting-summary-flat.pdf

Life sciences study design

All studies must disclose on these points even when the disclosure is negative.

Sample size	n = 7 rats per group; ~16 electrode contact sites per rat. Sample sizes were determined by power analysis at time of IACUC submission
Data exclusions	Electrode channels (contacts) were tested prior to implantation in the rats; Contacts that were malfunctioning prior to implantation were excluded from analysis.
Replication	Repeat measurements were performed for each of the experiments described.
Randomization	Animals were randomly assigned to one of four treatment groups.
Blinding	Investigators performing recording analysis were blinded to the treatment groups.

Reporting for specific materials, systems and methods

We require information from authors about some types of materials, experimental systems and methods used in many studies. Here, indicate whether each material, system or method listed is relevant to your study. If you are not sure if a list item applies to your research, read the appropriate section before selecting a response.

Materials & experimental systems

n/a	Involved in the study
☐	☒ Antibodies
☒	☐ Eukaryotic cell lines
☒	☐ Palaeontology and archaeology
☐	☒ Animals and other organisms
☒	☐ Clinical data
☒	☐ Dual use research of concern
☒	☐ Plants

Methods

n/a	Involved in the study
☒	☐ ChIP-seq
☒	☐ Flow cytometry
☒	☐ MRI-based neuroimaging

Antibodies

Antibodies used	Primary: GFAP, Dako Z033429-2; CD68, Sigma-Aldrich MAB1435; IgG, Bio-Rad, 618501; NeuN, Millipore Sigma MAB3477; Secondary: Invitrogen A11037, Invitrogen A11029
Validation	Primary and secondary antibody control slides were run to confirm the lack of non-specific staining. Validated by the manufacturers and used as received.

Animals and other research organisms

Policy information about studies involving animals; ARRIVE guidelines recommended for reporting animal research, and Sex and Gender in Research

Laboratory animals	Male Sprague Dawley, Charles River, CD, 250-280 g
Wild animals	n/a
Reporting on sex	Sex was not considered in this study. Our group has previously studied sex-balanced cohorts of mice and have not found statistically significant differences with respect to neuroinflammation in response to implanted NeuroNexus electrodes. https://doi.org/10.3389/fnins.2018.00772
Field-collected samples	n/a
Ethics oversight	All studies were reviewed and approved by the Institutional Animal Care and Use Committee (IACUC) at the Louis Stokes Cleveland Department of Veterans Affairs Medical Center.

Note that full information on the approval of the study protocol must also be provided in the manuscript.

Plants

Seed stocks	n/a
Novel plant genotypes	n/a
Authentication	n/a
